# Understanding the anatomy of ears from guinea pigs and rats and its use in basic otologic research

**DOI:** 10.1016/S1808-8694(15)30830-2

**Published:** 2015-10-18

**Authors:** Agnes Afrodite Sumarelli Albuquerque, Maria Rossato, José Antonio Apparecido de Oliveira, Miguel Angelo Hyppolito

**Affiliations:** 1 Bachelor’s degree in Biological Sciences, Lab technician at the Ophthalmology, Otorhinolaryngology and Head and Neck Surgery Department - Faculdade de Medicina de Ribeirão Preto - USP.; 2 Lab technician at the Ophthalmology, Otorhinolaryngology and Head and Neck Surgery Department - Faculdade de Medicina de Ribeirão Preto - USP.; 3 Full Professor, Head of the Ophthalmology, Otorhinolaryngology and Head and Neck Surgery Department - Faculdade de Medicina de Ribeirão Preto - USP.; 4 PhD, Professor - Ophthalmology, Otorhinolaryngology and Head and Neck Surgery Department - Faculdade de Medicina de Ribeirão Preto - USP.; Faculdade de Medicina de Ribeirão Preto da Universidade de São Paulo.

**Keywords:** guinea pigs, photomicrography, scanning electron microscopy, ear, rats

## Abstract

The use of animal samples is important in otologic research and understanding the anatomy of their ears help make proper use of them in research projects. **Aim:** to study guinea pig’s and rat’s ears under light microscopy(LM) and scanning electron microscopy(SEM) and understand their anatomical advantages in basic otologic research. **Materials and methods:** The temporal bones, tympanic bullas and cochleas from three albino guinea pigs and rats were photographed and analyzed under LM and SEM. **Results:** Rats aren’t as simple to handle as guinea pigs, and often present with otitis media. Rats have a fragile junction of the tympanic bulla, two and half turns in the cochlea, and their tympanic membranes do not seal off the entire external auditory canal. Guinea pigs have full bullas, their incus and malleus are fused and they have three and half cochlear turns. Under SEM, guinea pigs and rats have Tectori Membrane, Raissner’s Membrane and the Organ of Corti. Only guinea pigs have Hensen’s Cells. **Conclusion:** Guinea pigs were considered easy to handle for microdissection purposes because of the size and robustness of their temporal bones, and for surgical experiments involving the stapes, the oval window and the tympanic membrane. Under SEM there are similarities guinea pigs and rats, and both can be used in inner ear studies.

## INTRODUCTION

Otological research frequently requires the use of experimental models, mainly guinea pigs and rats because they are easy to handle and their ears are similar to those of humans. Although there are numerous otological studies with guinea pigs and rats, in our bibliographic survey we did not find enough information about the inner and outer ear anatomy of these animals.

Knowledge about the anatomy of the ears from these animals is paramount in the field of otology. A better use of these animals makes the results attained more reliable, avoiding the use of techniques which are not adequate for a certain species and, thus, causing unnecessary morbidity and mortality to a large number of animals.

According to Schanaider et al.^1^, most of the research in the medical field is undertaken with small size animals (mice, rats, hamsters, guinea pig or gerbil) and comprehend almost 90% of all the species of animals used in a laboratory.

According to Oliveira^2^, in his paper regarding the labyrinthine system, the guinea pig was chosen because its hearing and vestibular systems are very similar to those of humans, and it is an animal that is very good for experiments with the labyrinth.

Differently from that, Santos et al.^3^, used the rat as an experiment animal because it has practical advantages and because of the anatomical and pathological similarities between its ear and the human ear.

In order to facilitate the research of the investigators in the field of labyrinthology using guinea pigs as experimental animals, Oliveira^2^ described the anatomy of these animals’ temporal bones, with a dissection under stereoscopic view of the anatomic specimens and microphotographies of surface preparation from cochlear and vestibular epithelium. Such knowledge is essential, especially in the study of the structural alterations caused by drugs which are toxic to the labyrinth and acoustic trauma.

Judkins and Li^4^ described the topographic anatomy of the albine rat’s middle ear by means of microphotographies, illustrating different surgical exposures through structural details. Many of the middle ear’s anatomical structures of the albine rat exist in human beings, nonetheless, the facial nerve emerges from the temporal bone in a more superficial and antero-rostral position. The ossicles are thinner than the human ones, almost totally hidden in the epitympanic region, and the carotid artery courses between the stapes crura. They conclude that although dissection and exploration can be carried out in slaughtered animals, the research procedure is more frequently designed based on live animals that will later on be assessed in order to analyze some scientific assumption.

Pinilla et al.^5^ described a simple access to the rat’s middle ear causing minimum morbidity and mortality to the animals. When compared to the guinea pig, the rat’s middle ear anatomical structures are more similar to those from human beings. The only exception is the more superficial and antero-rostral location of the facial nerve in the rat when compared to guinea pigs and humans. However, the guinea pig’s cochlea is bigger and the ossicular chain is fused at the incus-malleus level, while that of the rat is projected to inside the ear, the ossicles are clearly defined and can be removed separately, as in humans. Rats are not good as a model for stapes surgery or ossiculoplasty involving ossicle replacement because of the carotid artery which courses along the base of the cochlea and through the stapes crura, in this case an exposure of the oval window can cause damage by hemorrhage, cochlear lesions and animal death. On the other hand, easy access to the round window and the lateral wall can be an alternative for cochleostomy without causing damage to the tympanic membrane or the ossicles.

They concluded that specific care is necessary when one is dealing with new accesses to the middle ear of small animals, such as rats, when one desires a long life and subsequent follow up.

The present investigation used Light Microscopy (LM) and Scanning Electron Microscopy (SEM) in order to compare middle and inner ear structures of the rat and the guinea pig and establish which animal has the better structures for each type of experiment.

## OBJECTIVES

To study the macroscopic and microscopic anatomy of the guinea pig and the rat’s ear by light microscopy (LM) and Scanning Electron Microscopy (SEM), seeking anatomical advantages for its use in experimental models in basic otologic research.

## MATERIALS AND METHODS

This study was submitted to the Ethics Committee in Animal Experimentation of the Medical School of Ribeirão Preto of the University of São Paulo, and approved under protocol # 078/2007.

For this study we used three albine guinea pigs (Cavia porcellus - English lineage) and three albine rats (Rattus norvegicus - Wistar Lineage) weighing between 250 and 300 grams.

The guinea pigs and the rats were anesthetized with Ketamin 50mg/ml - Cristalia Labs,) 40 mg/kg and Dopaser 20 mg/ml, Calier do Brasil Labs,) 10 mg/kg and then later they were slaughtered by beheading.

Their temporal bones with their tympanic cavities were quickly removed and opened for cochlear exposure.

The temporal bones were open with a dissection scissors, which was later one placed on the back of their necks, doing a median longitudinal cut on the skull going all the way reaching behind the ears. Afterwards, using the hands in the external auditory meatus as a guide, the tympanic bulla was located with the thumbs and through an outwards movement it was separated from the other structures; having it locked between the fingers it was twisted in a smooth movement to loosen the tissue and release it.

The bulla was opened by holding it with one of the hands and with a small forceps an opening was made in the posterior air sinus (mastoid) following through its notch until the tip of the petrous. Following that, placing the forceps in the external auditory meatus and anterior air sinus, in a single movement, it was broken and all the bony portion of the bulla was raised, thus exposing the cochlea.

The material was fixed by slowly injecting 3% glutaraldehyde, through an opening made on the cochlear apex and on the round window and remained in immersion for 4 hours at 4°C. The material was flushed many times in a 0.1M phosphate buffer solution and submitted to microdissection in order to expose the cochlear turns and the vestibular system.

The guinea pig’s and rat’s bullas were photographed with the help of a digital camera (Nikon - Coolpix 990, 3.34 mega pixels) coupled to a microscope (DF Vasconcelos).

The parts photographed were: the closed bullas and the cochleas with their bony coverage, the open bulla and the cochlea without its bony coverage, the tympanic membrane, the ossicles alone and the cochlea alone.

The cochleas from a guinea pig and a rat, in order to be analyzed through electronic scan microscopy, were fixed in 3% glutaraldehyde and flushed in 0.1M buffer solution, then later refixed in 1% osmium tetroxide solution and 0.1M phosphate buffer for 2 hours at 4°C, and then washed many times in 0.1M phosphate buffer and pH of 7.3.

Right afterwards the structures were dehydrated through ethanol baths in growing concentrations of 50, 70, 90 and 95%, for about 10 minutes each. After that, we used 100% ethanol in three 20 minute baths, and in the last bath the structures were left immerse at room temperature for 12 hours.

The water still present in the material after dehydration was removed during the drying process, carried out by the liquid carbon dioxide critical point method. The sample within a proper container was transferred to the critical point device’s drying chamber (BAL-TEC-CPD 030 - Critical Point Dryer) where, through successive baths with liquid carbon dioxide at 4°C, the ethanol was discarded. After completely removing the ethanol, the material was submitted to a temperature increase of up to 40°C, so as to have the carbon dioxide go from the liquid to the gaseous stage, and this only happens at 31°C - critical point.

With the dried material, we assembled the cochleas in a cylindrical metal specimen-holder (stubs) with carbon conductive paste. Afterwards it received a thin layer of gold applied with a BAL-TEC SCD 050 - Sputter Coater in order to become electrically conductive.

After finishing these processes, the guinea pigs’ cochleas and those of the rat had their structures studied by scanning electron microscopy. The microscope used was JEOL JMS 5200.

## RESULTS

We noticed that the rat is not as simple to handle as the guinea pig, it is not such a docile animal and also because of its smaller head the tympanic bulla is fragile, making its handling more delicate.

Comparing guinea pigs with rats, it was easier to develop otitis media in the latter (5 middle ears from 3 rats and 1 middle ear from 3 guinea pigs had otitis media). These infections make the bone stronger, and this makes it difficult to open the tympanic bulla for fixation and facilitates damage to other structures, such as the cochlea, vestibular system and ossicles.

### Light Microscopy

**Figure 1:**
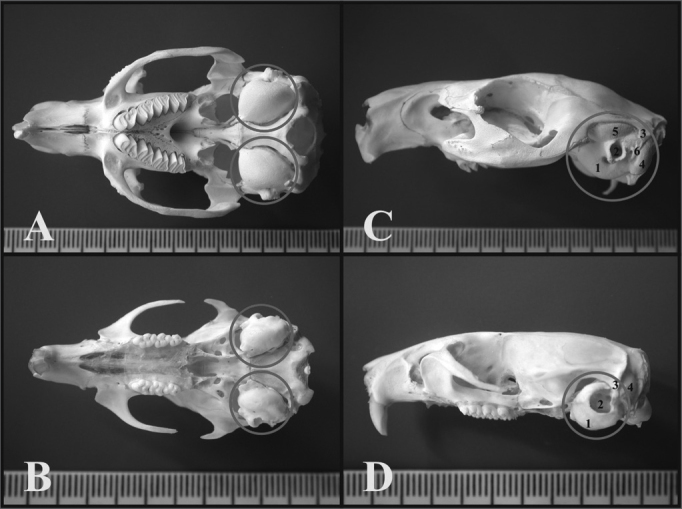
A) Guinea pig’s skull base showing the tympanic bullas (lower view) (millimeter scale); B) Rat’s skull base showing the tympanic bullas (lower view) (millimeter scale); C) Lateral left-side view of a young guinea pig’s skull (millimeter scale); D) Lateral left-side view of a young-adult rat’s skull - (millimeter scale). 1 - Bulla; 2 - External auditory canal; 3 - Squamous bone; 4 - Posterior air Sinus notch; 5 - Anterior air sinus; 6 - Para-occipital apophysis.

Through light microscopy it was possible to see that the guinea pig and the rat bullas were located in the skull’s postero-inferior region and only the petrous portion and the tympanic bone were joined. In the upper portion of the guinea pig’s bulla, the squamous bone forms a long process that continues ahead to make up the zygomatic arch (Figure 1).

Through microdissection under light microscopy to open the tympanic bullas, it was easier to do it with the rats’, because both parts of the bulla had a weaker joint. This does not happen with the guinea pig, which has a full and tougher tympanic bulla.

**Figure 2:**
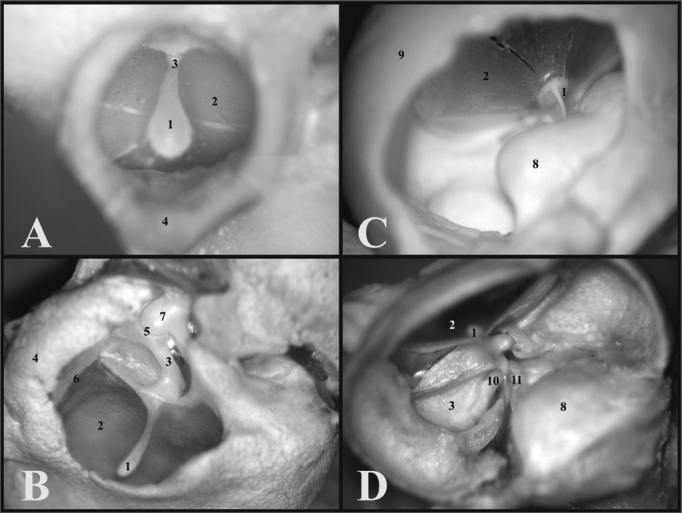
A) View through the external auditory canal of a young guinea pig (microphotography, 60X magnification); B) View through the external auditory canal of a young-adult rat (microphotography, 75X magnification); C) Inside view of the auditory cleft in a young guinea pig (inside the left tympanic bulla) (microphotography, 58X magnification); D) Inside view of the auditory cleft of a young-adult rat (inside the left tympanic bulla) (microphotography, 75X magnification). 1 - Malleus handle; 2 - Tympanic membrane; 3 - Malleus (neck); 4 - External auditory canal; 5 - Incus-malleus joint; 6 - Tympanic ring; 7 - Incus; 8 - Cochlea with bony cover; 9 - Bulla; 10 - Long process of the incus; 11 - Stapes.

The guinea pig’s inner auditory meatus is smaller than that of the rat, allowing one to see only the tympanic membrane and the malleus handle, while in the rat one can see the tympanic membrane which does not seal off the entire external auditory canal and the ossicular chain (Fig.2).

The cochlear insertion is more outward and bigger than that of the rat, allowing one to see the tympanic membrane, the malleus handle and the cochlea through the bony opening, because the other ossicles are covered by the cochlea. Now, in the rat, since the cochlea is smaller and its insertion is lower, it is possible to see the tympanic membrane, the malleus handle, the incus and part of the stapes (Fig. 2).

**Figure 3:**
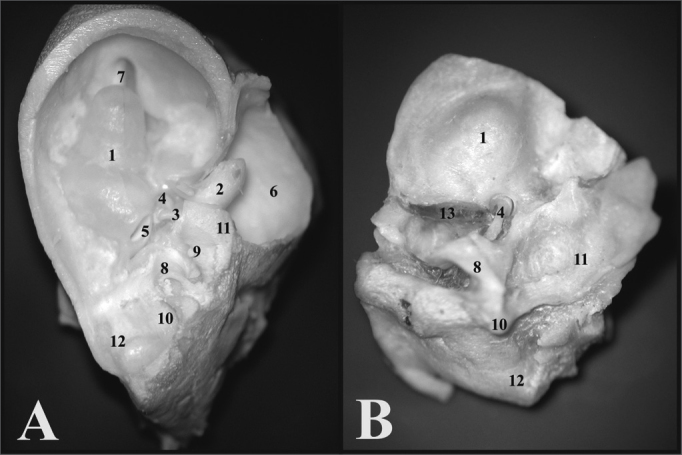
A) View inside the tympanic bulla of a young guinea pig with ossicles and cochlea with the bony cover (microphotography, 15X magnification); B) View inside the tympanic bulla of the young-adult rat with the stapes and the cochlea with the bony cover (microphotography, 28X magnification). 1 - Cochlea; 2 - Malleus; 3 - Long process of the incus; 4 - Stapes footplate over the oval window (stapes); 5 - Round window; 6 - Mastoid; 7 - Eustachian tube opening; 8 - Lateral semicircular canal projection; 9 - Facial nerve canal bony projection; 10 - Posterior semicircular canal projection; 11 - Superior semicircular canal projection; 12 - Posterior air sinus projection; 13 - Carotid artery.

When one opens the guinea pig’s tympanic bulla, the cochlea is seen inside, with its bony cover and the ossicular chain, thus it is possible to notice only the round window, since the oval window is covered by the stapes. In the rat, when one removes the upper portion of the bulla, the ossicles, malleus and incus come out together with the tympanic membrane, and inside the only remains are the cochlea with its bony cover and the stapes on the oval window. The round window can not be seen, because it is covered by the carotid artery which, in the rat passes between the stapes crura (Fig. 3).

During microdissection, when the bony cover of the guinea pig’s and rat’s cochleas is removed in order to expose it, it is not possible to keep the ossicles, and the oval window is opened.

**Figure 4:**
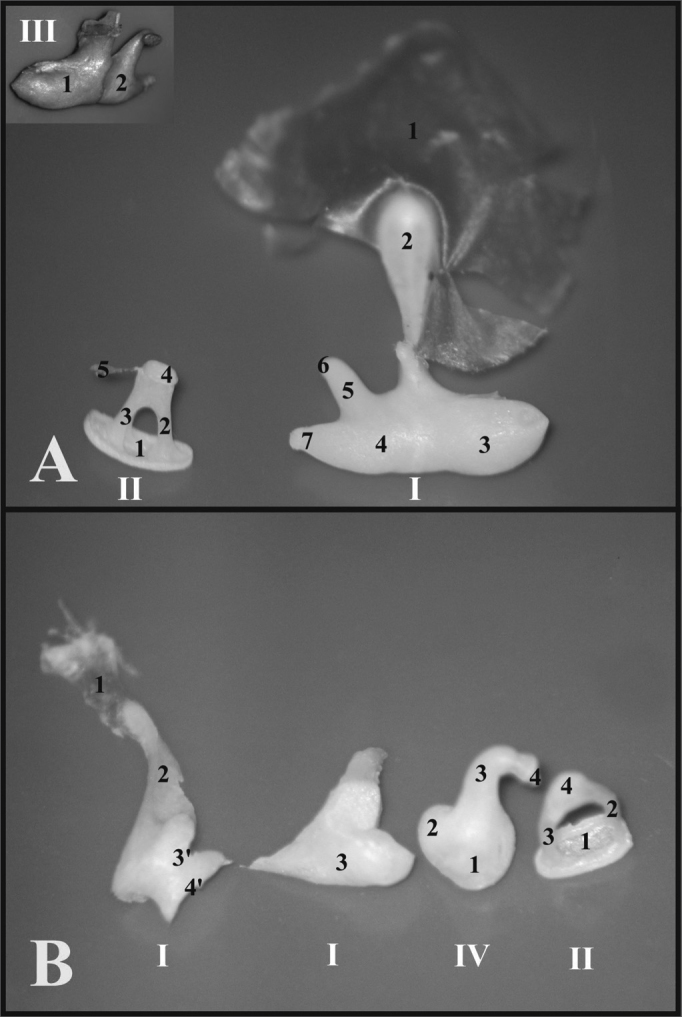
A) Set of ossicles of a young guinea pig. Detailed view of the malleus-incus joint (III) (microphotography, 58X magnification); B) Set of ossicles from a young-adult rat (microphotography, 60X magnification). I - Malleus - Incus 1 - Tympanic membrane; 2 - Malleus handle; 3 - Head of the malleus; 3’ - Neck of the malleus; 4 - Incus (fused to the malleus); 4’ - Joint surface of the malleus-incus; 5 - Long process of the incus; 6 - Lenticular process of the incus; 7 - short process of the incus II - Stapes 1 - Footplate of the incus; 2 - anterior crus of the stapes; 3 - Stapes posterior crus; 4 - Stapes Capitulum; 5 - Stapes muscle tendon III - Malleus-incus joint 1 - Malleus; 2 - Incus IV - Incus 1 - Incus-Malleus joint surface; 2 - Short process of the incus; 3 - Long process of the incus; 4 - Lenticular process of the incus.

As far as the ossicles are concerned, in the guinea pig, the malleus and the incus are fused, forming a junction called incus-malleus, while in the rat these are not fused. The guinea pig’s stapes is larger and has a triangular shape, in the rat the anterior and posterior crus are thinner and have a roundish shape (Fig. 4).

In the guinea pig, the saccule is thinner and is inserted close to the cochlear basal turn, and in the rat it is wider and inserted near the foot of the second basal turn.

**Figure 5:**
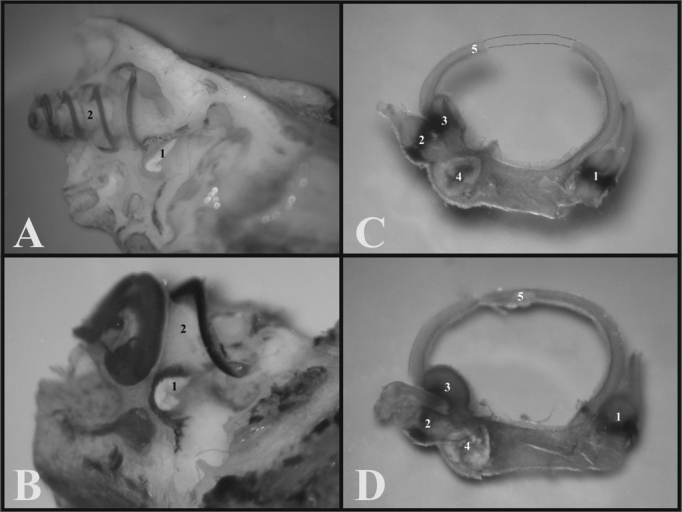
A) Internal wall of the left labyrinth from a young guinea pig showing the vestibule (microphotography, 55X magnification); B) Internal wall of the left labyrinth of a young-adult rat, showing the vestibule (microphotography, 60X magnification); C) Left membranous labyrinth of a young guinea pig (microphotography, 48X magnification); D) left membranous labyrinth from a young-adult rat (microphotography, 48X magnification). A and B) 1 - Saccule 2 - Cochlea C and D) 1 - posterior semicircular canal ampulla; 2 - superior semicircular canal ampulla; 3 - Lateral semicircular canal ampulla; 4 - Utricle; 5 - Lateral semicircular canal

The guinea pigs’ and rats’ ampullae are similar in appearance, however proportional to the animals’ sizes. The rats’ utricle is more easily removable than that of the guinea pig (Figure 5).

**Figure 6:**
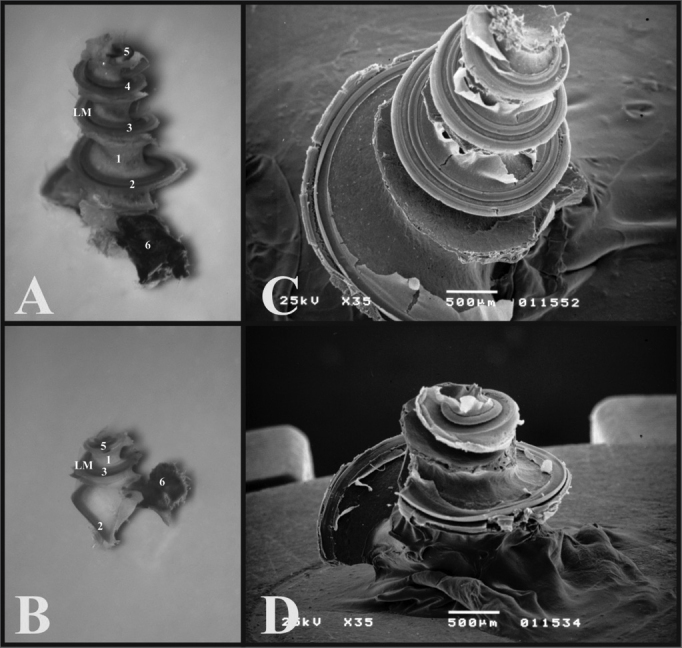
A) Cochlea from a young guinea pig, showing the cochlear turns with the membranous labyrinth (ML) (Microphotography, 44X magnification); B) Cochlea from a young-adult rat, showing the cochlear turns with the membranous labyrinth (ML) (Microphotography, 44X magnification); C) Guinea pig cochlea seen under scanning electron microscope (scanning electron micrograph, 35X magnification); D) Rat cochlea seen under scanning electron microscopy (scanning electromicrography, 35X magnification). 1 - Modiolus; 2 - Basal turn (E1); 3 - Turn 2 (E2); 4 - Turn 3 (E3); 5 - Apical turn; 6 - Cochlear nerve.

The guinea pig’s cochlea has three and one half turns, while that of the rat has two and one half. The rat’s basal turn is tougher and fused to the cochlear bony cover that is why often times during microdissection, when the bony cover is removed, the turn comes off with it (Fig. 6).

### Scanning Electron Microscopy

With scanning electron microscopy, the cochlear structures were analyzed in greater details and it was possible to see the three and one half turns of the guinea pigs’ cochleas and the two and one half of the rats’. Guinea pigs have the basal turn, the second turn, the third turn and the apical turn, while rats have the basal turn, the second and the apical (Fig. 6).

In all the turns from the cochleas of the guinea pigs and rats, we see the tectorial membrane, the Raissner’s membrane, the Organ of Corti and Hensen’s Cells.

The tectorial membrane is similar for both rats and guinea pigs. In preparing the cochlea for scanning electron microscopy, this membrane shrinks, detaching itself from the outer hair cells with its tip turned upwards.

Raissner’s membrane is also similar in guinea pigs and rats. It has two faces, the endolymphatic and the perilymphatic. In the guinea pigs and in rats the endolymphatic face is smoother and the perilymphatic has some cells with their nuclei standing out.

**Figure 7:**
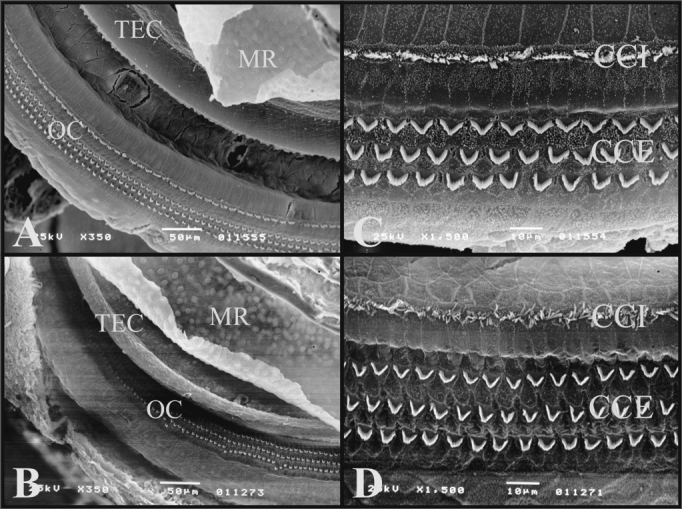
A) Guinea pig cochlea turn showing the Raissner’s Membrane (RM), Tectorial Membrane (TEC), Organ of Corti (OC) (Scanning electromicrography, 350X Magnification (TEC), Organ of Corti (OC) (Scanning electromicrography of the cochlear basal turn with the outer hair cells (CCE) and inner hair cells (CCI) (scanning electron microscopy, 1500X magnification); D) Organ of Corti from the basal turn of the rat’s cochlea with the Outer Hair Cells (CCE) and Inner Hair Cells (CCI) (scanning electron microscopy, 1500X magnification).

The Organs of Corti of both guinea pigs and rats are similar in the W/V pattern of the outer hair cells (OHC) and the inner hair cells (IHC). The OHC of the third roll in rats have cells out of the roll at each space interval (Figure 7).

In the basal turn of the guinea pig and rat cochleas, the W/V pattern and the OHC and IHC are equally arranged (Fig. 7).

On the 2nd turn, the third roll of OHC do not have a well-defined W/V pattern in guinea pigs and rats, these cells vary in their positions.

The third cochlear turn is only present in guinea pigs and it has second and third rolls without a well-defined W/V pattern, some may look like a half-moon.

The apical turn in both guinea pigs and rats have an OHC and IHC disarray regarding the W/V pattern and in regards of the roll of cells which is not well defined in rolls 2 and 3.

**Figure 8:**
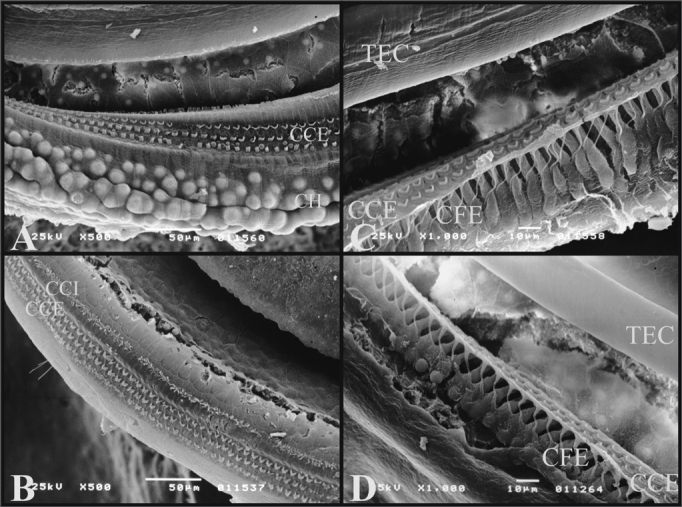
A) 3rd cochlear turn of the guinea pig showing the Hansen Cells (CH) (scanning electron micrograph, 500X magnification); B) 2nd cochlear turn from a rat, showing the lack of Hansen Cells definition (Scanning electron microscopy, 500X magnification); C) Guinea pig’s cochlea basal turn showing the external phalanx cells (CFE) (Deiters) (scanning electron micrography, 1000X magnification); D) Rat’s cochlea basal turn showing the external phalanx cells (CFE) (Deiters) (Scanning electron micrography, 1000X magnification).

On the guinea pigs’ turns, it is possible to see the well-defined Hensen’s Cells, and they are not present in rats (Fig. 8).

It was possible to see the external phalanx cells (Deiters) which support the OHCs. They are similar both in guinea pigs and rats.

## DISCUSSION

In otological research, the animals which are more frequently used are the guinea pigs and the rats because their ears are similar to those of humans.

According to the goal of each experiment we chose one species of animal.

The present study has shown that during the microdissection stage, the best animal to handle is the guinea pig, because of its size and the strength of the temporal bone which allow for a better removal of the tympanic bullas, while in the rat it is smaller and there is a greater likelihood of breaking the tympanic bullas at the time of their removal.

Through light microscopy it was possible to see the diameter of the guinea pig’s external auditory canal, which is smaller than that of the rat; however, the guinea pig’s tympanic membrane is much larger than the tympanic ring diameter, in such a way that it creates a hidden space anteriorly to the membrane. Thus, studies regarding the tympanic membrane can be better developed in the guinea pig.

In the rat’s tympanic membrane there is an opening between the incus and the malleus which accesses the middle ear. This opening can be the cause of otitis media in these animals.

When the study’s goal is to collect data with the ossicles intact, the best animal is the guinea pig, because when the tympanic bulla is opened the ossicles are not damaged. Contrary to that, in rats, the ossicles are stuck to the tympanic membrane, and then when the bulla tympanica is opened, the ossicles are removed together with the membrane.

Because of the malleus-incus process which is formed by the junction between the malleus and the incus - which are fused in guinea pigs, it makes it difficult change only one of these ossicles. However, if the goal of the procedure is the stapes surgery or the administration of some drug through the oval or round window, the best adapted animal is the guinea pig, and rats must be avoided because their carotid arteries pass between the stapes crura and remain next to the round window.

As shown before, the guinea pig cochlea has three and a half turns and that of the rat has two and a half, therefore, when one tries to test some drug that will have its effect in the cochlea, the best animal for that is the guinea pig.

With scanning electron microscopy, it was possible to notice that as to the number and appearance of the OHC and IHC both animals are similar, with a difference in the number of turns, and rats have one less than the guinea pig.

Another difference noticed was the presence of Hansen Cells which were only observed in the guinea pigs’ turns, therefore when the study is for this cell-type, the best animal is the guinea pig.

The OHC disarray of the third cochlear roll in rats does not seem to interfere when the experiment is carried out with a drug which causes this effect, because this disarray is continuous.

Since most cochlear structures are similar under scanning electron microscopy, it is possible that studies which use this method of analysis be carried out both in rats and in guinea pigs.

## CONCLUSIONS

After the stages of microdissection, we observed that the best animal to handle is the guinea pig.

According to the analyses carried out under light microscopy it is possible to see that for experiments involving stapes surgery and cochleostomy, one must avoid rats as experimental animals, and in surgeries that approach the tympanic membrane, the most proper animal is the guinea pig.

Analyses through scanning electron microscopy showed that the cochlear structures of the guinea pig and rat are similar, thus both can be used in experiments that approach it.

Therefore, for otological studies, the guinea pig is a good lab animal, with some advantages; however, rats can also be used, as long as the care hereby discussed is taken.
